# Wheat mycobiome dynamics driven by interseasonal crop-crop transfer and Fusarium head blight

**DOI:** 10.3389/fmicb.2026.1778987

**Published:** 2026-03-04

**Authors:** Briana K. Whitaker, Kristi Gdanetz, Martha Marie Vaughan, Susan McCormick, Talon Becker

**Affiliations:** 1 United States Department of Agriculture, Agricultural Research Service, National Center for Agricultural Utilization Research, Mycotoxin Prevention and Applied Microbiology Research Unit, Peoria, IL, United States; 2 United States Department of Agriculture, Agricultural Research Service, Cereal Crops Research Unit, Madison, WI, United States; 3 Department of Crop Sciences, Illinois Extension, University of Illinois at Urbana-Champaign, Champaign, IL, United States

**Keywords:** corn-wheat rotation, debris, foliar endophytes, fungal microbiome, *Fusarium* graminearum, mycotoxin, phyllosphere, vomitoxin

## Abstract

**Introduction:**

Fusarium head blight (FHB) is a devastating disease of wheat that causes mycotoxin contamination in grains. Diseases like FHB have traditionally been managed with integrated strategies; but this has led to a proliferation of fungicide-resistant pathogens and soil erosion while full disease control has remained elusive. Leveraging the microbiome for more sustainable management is an alternative, however, translation of promising strategies is hampered by our limited understanding of crop microbiome differences across plant development and tissue types.

**Methods:**

We characterized fungal communities using amplicon sequencing across five developmental timepoints in wheat leaves and wheat heads, as well as in maize debris from the previous growing season. Samples were collected from two locations in Illinois, USA. We assessed how tissue type, site, developmental stage, and wheat variety contributed to mycobiome composition. Source–sink relationships among debris, leaves, and heads were evaluated, and taxa associated with high and low FHB conditions were identified. Network analyses were used to determine the roles of key fungal taxa in wheat head and maize debris microbiomes.

**Results:**

Mycobiome composition varied strongly by tissue type, though site and developmental timepoint were also important contributors. Host variety conditionally explained mycobiome variation in wheat heads, but not in leaves or debris. We also identified debris as a major fungal source to leaves early in development, but not later—and found that leaves were never a large inoculum source to head mycobiomes at either developmental stage tested. Taxa enriched under high FHB conditions in wheat heads belonged to the Ascomycota (*Cladosporium*, *Pseudopithomyces*), while taxa enriched under low FHB conditions primarily belonged to the Basidiomycota (*Filobasidium, Sporobolomyces, Tilletiopsis, Entyloma*). Fusarium spp. were important nodes in wheat head and maize debris microbiome networks.

**Discussion:**

This work shows that fungal movement from crop to crop across seasons, and between plant tissues within a season, shape phyllosphere microbiome dynamics and can indicate potential disease outcomes in the FHB pathosystem. As microbiome-based disease management develops alongside rapid growth in the biologicals industry and increased recognition of microbial roles in agriculture, this work highlights several promising directions. These include identifying basidiomycetous yeasts associated with low FHB, pinpointing taxa correlated with Fusarium in wheat heads and maize debris, and demonstrating that applying biocontrols to wheat leaves is unlikely to affect pathogen spread to heads. Future research should focus on controlled tests of microbe-microbe interactions and their impacts on plant immunity, disease suppression, and yield.

## 1 Introduction

Wheat (*Triticum aestivum* L.) is one of the most widely cultivated crops in the world and a critical source of calories, protein, and nutrition for the globe’s population (Shewry and Hey, 2015). One of the most important diseases of wheat is *Fusarium* head blight (FHB) caused by members of the fungal genus *Fusarium*, which colonize wheat heads during flowering (Shah et al., 2013). FHB is a devastating wheat disease because it not only causes yield losses due to reduced grain fill, but also because *Fusarium* spp. produce toxic secondary metabolites (i.e., mycotoxins) inside the grain producing food safety risks for humans, livestock, and pets (Wegulo et al., 2015; Chen et al., 2019). These mycotoxins are chemically diverse with equally variable health impacts, including vomiting, food refusal, compromised immune function, and reduced fertility in humans and livestock (Gurikar et al., 2023). In the USA, *F. graminearum* and deoxynivalenol (DON or vomitoxin) are the most prevalent causal agent of FHB and economically important toxin of concern, respectively (Ward et al., 2008; McMullen et al., 2012).

Despite advances to integrated management practices for FHB and the availability of moderately resistant wheat cultivars, complete control of the disease continues to be elusive (Shah et al., 2017; Miedaner et al., 2023), and is being further impacted by changing environmental norms, sustainability practices, and policies surrounding pesticide regulations (Ejaz et al., 2023). Fortunately, the sequencing revolution has created new opportunities to harness the genetic flexibility of the microbiome for improved crop health (Compant et al., 2024). The scientific community has identified three potential intervention strategies utilizing the crop microbiome: (1) application of microbial strains as biocontrols, plant growth stimulants, or fertilizer replacements (Santos et al., 2021; Stenberg et al., 2021; Martinez et al., 2024); (2) adaptive breeding strategies with more beneficial microbiome compositions as the target (Wallace et al., 2018; Spor et al., 2020; Wagner, 2021); and (3) integrative management practices that support healthier, more resilient microbiomes (Mo et al., 2024; Seitz et al., 2024). However, effective translation of these approaches to farms will require an understanding of the pathology and ecology of the plant microbiome, including its pathogenic members, over the course of plant development and across different tissue types.

Aboveground plant (hereafter phyllosphere) microbiomes are dynamic communities that experience rapid, successional change over the course of plant development (Shade et al., 2013). New leaves and reproductive tissues emerge from elongating stems or tillers, which present novel colonization environments where successional dynamics can drive microbiome assemblage (Dibner et al., 2021). Additionally, the host immune system does not remain static. Important transitions in plant immune control of microbial colonizers occur early in development (Paasch et al., 2023), as well as during the reproductive phase during which microbiome co-occurrence networks often exhibit enhanced complexity and stability (Liu Q. et al., 2023; Fu et al., 2024; Sahil et al., 2025).

Microbial kingdom, tissue type, and exposure to different physical environments may also act as selective filters on community assembly processes in the phyllosphere. For example, leaf bacteria have been shown to be heavily sourced from soils in biofuel grasses (Grady et al., 2019) and the air in cereal crops (Xiong et al., 2024), while leaf fungi of grasses may be more closely sourced from leaves in the surrounding plant community (Latz et al., 2021; Whitaker et al., 2022). Tissue specificity is also a common feature in phyllosphere microbiome studies (Cregger et al., 2018; Longley et al., 2020), which may be driven in part by microbial traits, such as fungal spore size and dispersal mechanism (Gdanetz et al., 2021). In agricultural systems, the role of successional dynamics and microbial sourcing to community assembly may be particularly relevant for identifying which microbial taxa are most likely to interact with a pathogen in its target infection organ and during its optimal infection window. For example, *Puccinia* rust epidemics have major impacts on leaf and stem communities during late vegetative stages in barley (Sapkota et al., 2023), while FHB disease onset can only begin to impact the head microbiome after anthesis (Comby et al., 2016).

In agricultural systems, debris (e.g., litter, residue) from the previous crop is a well-known source of pathogen inoculum during the succeeding season (Lipps, 1988; Karlsson et al., 2021). *F. graminearum* can cause disease in both wheat and maize (*Zea mays*) hosts (i.e., FHB and Gibberella ear rot, respectively) and can overwinter as a saprophyte producing perithecia on decaying maize stalks (Khonga and Sutton, 1988). Thus, for US producers the increasing adoption of no- or low-till practices, frequency of maize-wheat rotations, and continued expansion of maize growing regions north- and west-ward has increased the risk landscape for FHB epidemics (Dill-Macky and Jones, 2000).

However, debris hosts other microbiota besides pathogens and it is unclear how the debris microbiome of the previous crop influences the succeeding crop’s microbiome and its resistance or susceptibility to *Fusarium* infection (Kerdraon et al., 2019). Importantly, the debris microbiome experiences successional dynamics similar to green tissue. For example, the debris microbiome gradually transitions over the course of the winter season (3–9 months) from a community that looks like the pre-senescence, green tissue microbiome to one that more closely resembles the soil community (Bastian et al., 2009; Voriskova and Baldrian, 2013). Additionally, while direct evidence for debris microbe recolonization onto germinating or re-greening plants is limited, the presence of debris in the local environment alters fungal leaf communities in cacao seedlings (Christian et al., 2017) and debris microbiota can induce fitness consequences in young flowering plants (Zaret et al., 2021). Alternatively, physical interactions between debris microbiota and the next season’s pathogens may indirectly reduce inoculum potential. For example, *Fusarium* spp. residing in maize debris were shown to be negatively correlated with *Vishniacozyma* and *Epicoccum* fungi (Cobo-Diaz et al., 2022). Similarly, *Aureobasidium* sp. and *Clonostachys rosea* strains derived from debris have been proposed as biocontrols of the chickpea pathogen *Didymella rabiei* (Dugan et al., 2005). These interseasonal dynamics remain important questions to address as environmental change impacts debris decomposition rates, crop disease risk, and microbial communities (Wahdan et al., 2022).

In this study, the main goal was to identify how mycobiome colonization in the wheat phyllosphere changes across tissue types and over the course of the growing season, as well as how the mycobiome responds to FHB disease and the presence of *Fusarium* pathogens. Specifically, we planted three winter wheat varieties into two USA Illinois fields previously planted to maize. The maize-wheat rotation is common in the midwestern USA and is known to increase risk for FHB onset and mycotoxin contamination (McMullen et al., 2012). Using this experimental pathosystem and fungal amplicon sequencing, we set out to address four hypotheses. (1) We hypothesized that tissue type and field location would be the primary drivers of fungal phyllosphere (leaves and heads) colonization based on previous work (Giauque and Hawkes, 2016; Carper et al., 2018; Wei and Ashman, 2018), but that host variety, developmental stage, and FHB disease status would still play a significant role in structuring the mycobiome. (2) Additionally, our study design incorporated three host varieties varying in their FHB resistance. Therefore, we also predicted that host variety would play a role in structuring the mycobiome, but that the importance of host variety would vary across tissue types. (3) We hypothesized that maize debris would be a moderate source of fungal colonizers to the wheat leaf community based on close physical proximity of the tissues during early plant development, and that likewise the upper canopy leaf communities would be major contributors to the wheat head communities due to tissue type similarity and proximity. (4) Lastly, plants experiencing biotic stress may recruit beneficial microbes (Liu et al., 2021) or colonization by beneficial microbes may reduce the likelihood of disease onset (Jiang et al., 2022). Therefore, we predicted that different suites of fungi would be recruited under low disease or associated with reduced *Fusarium* abundance.

## 2 Materials and methods

### 2.1 Winter wheat planting and experimental design

**FIGURE 1:**
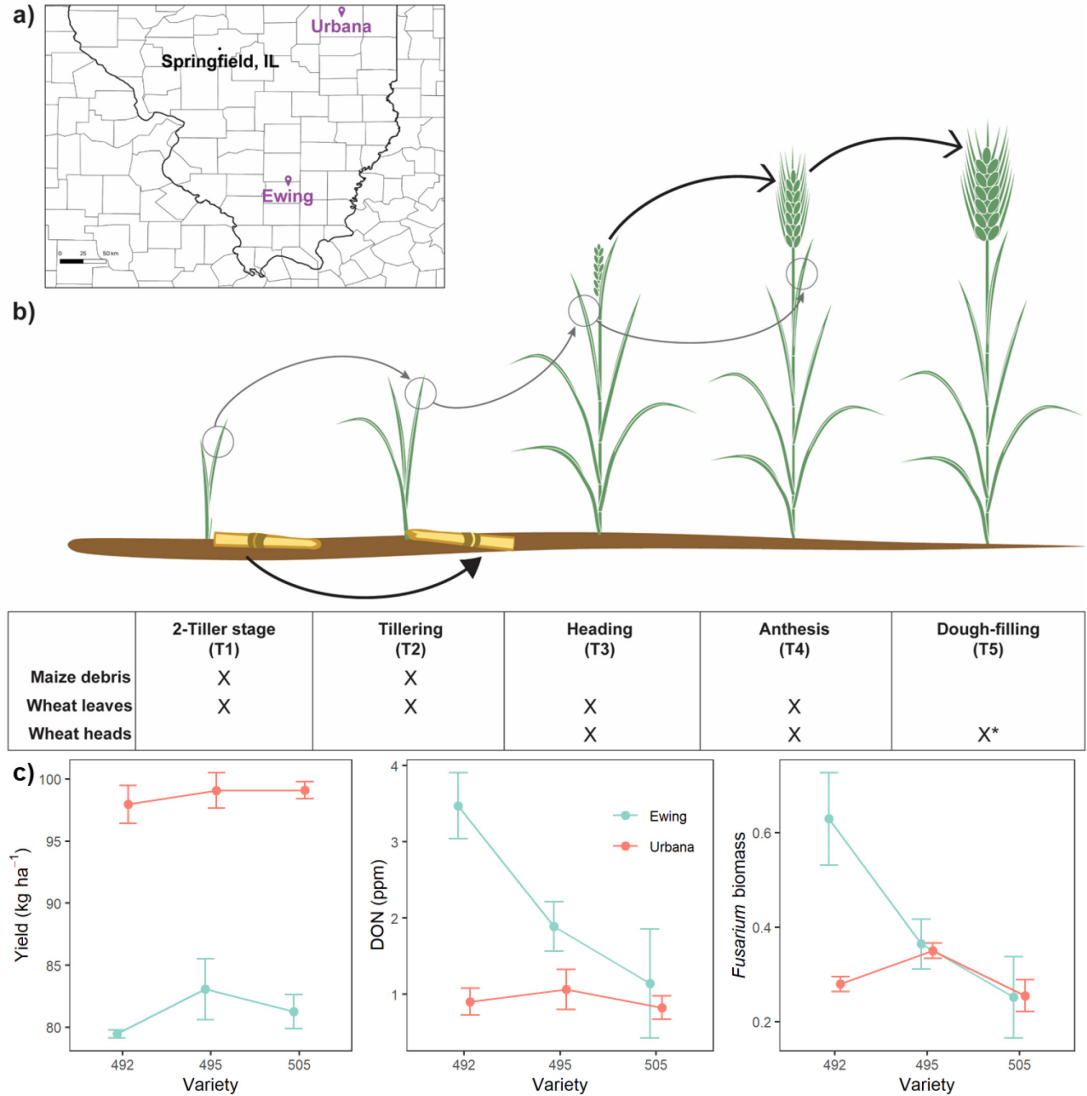
Field locations, temporal sampling strategy, and agronomic data by location. **(a)** Location of the two experimental field sites within Illinois, USA. **(b)** Wheat phyllosphere and maize debris samples were collected from five developmental timepoints (T1-T5). Image depicts growth of wheat plants over time, with verbal descriptions of developmental stages provided in the table. The table indicates the tissue types sampled at each timepoint. (*) For the wheat heads at T5, two head types were collected: low and high Fusarium head blight disease. Arrows represent potential between-timepoint transfers of microbiota as measured by a source-sink analysis. **(c)** Variation in agronomic factors across AgriMAXX varieties and locations. Data shows the mean and standard error for each treatment combination.

In the autumn of 2020, seeds of three soft red winter wheat varieties were planted into maize debris at two field sites in Illinois (Ewing: 38.096440, −88.845311; Urbana: 40.080776, −88.224160; Figure 1a) which were located over 225 km apart. At each site, a randomized complete block design was used where each of the four blocks contained one replicate plot each of all three varieties (i.e., 2 sites × 4 blocks ×x 3 varieties). The varieties planted were all AgriMAXX Wheat Co. varieties (492, 495, and 505; Mascoutah, IL), which have FHB resistance ratings of 5, 8, and 9, respectively (AgriMAXX internal scale: 1 = susceptible, 10 = resistant). Following conventional practices for the region, all seeds were treated with broad-spectrum pesticides: sedaxane, difenoconazole, mefenoxam, and thiamethoxam.

Given that our study aimed to evaluate fungal communities under realistic field conditions, the agronomic practices and field plot sizes were not standardized across sites. Instead, agronomic practices for each region were followed and local planting choices were selected based on site differences in weather, climate, and soil type (Supplementary Table S1). Full management details are provided in Supplementary Table S1. Briefly, neither Ewing nor Urbana was tilled, and maize debris was visibly present on the soil layer prior to planting. The seeding rate was 145.7 kg ha^–1^ in Ewing and 160.3 kg ha^–1^ in Urbana. Plot dimensions were 6.1 m by 30.5 m in Ewing and 9.1 m by 35.4 m in Urbana, with filler wheat along site edges. To allow for natural FHB establishment, fungicides were not applied during either the vegetative or reproductive stages. Conventional fertilization practices for each region were followed.

The Ewing field site was harvested on June 24, 2021 after dry-down using a Gleaner K2. Due to poor stand density across some plots caused by high soil moisture, 4.0 m was removed from the north end of all plots (0.0161 ha plot −^1^). Whole plot yields were taken with a weigh wagon, while moisture content and test weight were calculated using a Dickey John mini-GAC (Auburn, IL). The Urbana field site was harvested on July 5, 2021 using an Almaco SPC 20 (Nevada, IA) with a HarvestMaster H2 Grain gauge for continuous weighing and moisture content measurements. In Urbana, two 1.5 m x 15.2 m strips from uniform sections of each plot were used to calculate yield, moisture, and test weight (0.00465 ha plot^–1^).

### 2.2 Mycobiome sample collection

Sample collection for mycobiome analysis targeted three tissue types (maize debris, wheat heads, wheat leaves) and five key developmental and seasonal changes in winter wheat growth, as well as key points for FHB disease pathogenesis (designated T1-T5; Figure 1b). Specifically, we sampled during: (T1) pre-winter tillering (Feekes 2); (T2) post-winter pseudo-stem erection (Feekes 4–5); (T3) heading (Feekes 10.1–10.5); (T4) flowering, when *F. graminearum* infection occurs (Feekes 10.5.1–10.5.3); and (T5) dough fill, when FHB symptoms present (Feekes 11.2–11.3).

For each tissue type and time point combination, either eight (leaf and debris) or four (heads) samples were purposefully collected to span the entire length and width of a field plot and then combined into one sample in sterile plastic bags (see Supplementary methods for details). Whole wheat leaves were harvested across the first four time points (T1-T4), typically targeting the second-most fully elongated leaf—to balance the need for leaf maturity with sufficient time since emergence for fungal colonization—except at T4, where the flag leaf was sampled as the ultimate leaf. Entire wheat heads were collected after emergence at the final three time points (T3–T5). For the T5 timepoint, two sets of heads were collected: heads displaying either low (<30%) or high ( > 30%) visual FHB symptoms. Lastly, maize debris was sampled at the first two time points (T1-T2), where the focus was a piece of decaying maize stem inclusive of a node. Scissors and gloves used for collection were sterilized with 70% ethanol between plots. Bagged samples were placed on ice for transport back to the lab and then frozen within 1 day of collection.

### 2.3 Illumina sample preparation

Leaf samples from each plot were randomly subsampled into 2 mm^2^ fragments. Forty-eight fragments per plot per time point were then surface sterilized by successive washes in 95% ethanol (30 s), 0.5% NaOCl (2 min), 70% ethanol (2 min), and sterile water (30 s), placed into a sterile tube, and flash frozen. DNA was extracted from leaf fragments using the Synergy 2.0 Plant DNA kit (OPS Diagnostics LLC, Lebanon, NJ). Head samples were processed as is by lyophilizing, grinding to a powder using a tissue homogenizer (1,650 rpm for 8 min, Geno/Grinder 2010; Cole Parmer, Vernon Hills, IL), and extracting DNA from ∼20 mg of tissue using the Synergy 2.0 Plant DNA kit. Debris samples were lyophilized, ground in three passes through a Stein Mill (Steinlite Co., Atchison, KS) for 10 s each, and DNA extracted in duplicate from 20 to 40 mg of ground tissue using the DNeasy PowerSoil kit (Qiagen, Germantown, MD). Debris DNA was pooled by plot prior to PCR amplification.

DNA concentration was fluorometrically quantified (Quant-It dsDNA kit, Thermo Fisher Scientific, Waltham, MA) on a Synergy H1 microplate reader (Agilent, Santa Clara, CA) and normalized to 10 ng μL^–1^. Gene amplification of fungal DNA was performed using two-stage PCR with the fITS7 (5′-GTGARTCATCGAATCTTTG-3′; Ihrmark et al., 2012) and ITS4-ngs (5′-TCCTSCGCTTATTGATATGC-3′; Tedersoo et al., 2014) primers modified with Illumina adapters (Smith and Peay, 2014). Primers were frameshifted using 3–6 random nucleotides to increase sequence complexity (Lundberg et al., 2013). Negative PCR controls were included at each stage of amplification. The first stage of PCR consisted of 12.5 μL 1 × Phusion High Fidelity PCR Master Mix (Thermo Fisher Scientific), 0.2 μM of each primer, 0.5 mg mL^–1^ BSA, and 25 ng DNA template. In addition, a custom-designed peptide nucleic acid (PNA) was added at 0.75 μM final concentration to the PCR reaction to block host genomic ITS amplification (5′-CAAGTTGCGCCCGA-3′; Whitaker, 2025) with PCR-grade water up to 25 μL total reaction volume. Thermocycling conditions were 30 s at 98°C, then 25 cycles of denaturing for 10 s at 98°C, PNA annealing for 10 s at 74°C, primer annealing for 30 s at 58°C, and extension for 30 s for 72°C, and a final extension for 5 min at 72°C. PCR reactions were cleaned using AMPure XP beads (Beckman-Coulter Inc., Indianapolis, IN). Each index PCR reaction consisted of 25 μL 1 × Phusion High Fidelity PCR Master Mix, 5 μL each of two indices (Nextera XT Index Kit v2, Illumina Inc., San Diego, CA), 5 μL DNA template, and 10 μL water. All products were cleaned using AMPure XP beads, quantified in triplicate using Quant-It on a Synergy H1 Microplate reader, and PCR fragment size determined using Tapestation (D1000 ScreenTape, Agilent Technologies, Santa Clara, CA). Lastly, all samples were pooled in equimolar ratios and sequenced on an Illumina MiSeq platform (v3 chemistry, 2 × 300 bp). Raw sequencing data are provided in the NCBI SRA BioProject PRJNA1297200, accession numbers SRR44969627–SRR44969870.

### 2.4 Disease and yield quantification

At the final timepoint, an additional set of wheat heads was collected to determine disease at the plot level. Specifically, 32 bulk wheat heads were randomly collected from eight points spanning the length and width of the field plot (with 4 heads collected per point) in order to avoid bias in FHB disease state and best represent microclimate differences across the plot. For these bulk wheat heads, we sampled heads irrespective of disease state or tiller height. The bulk wheat heads were lyophilized, hand threshed to remove chaff and stems, and the grains ground to a flour-like consistency using a Stein Mill. In between samples, the mill cups were cleaned with 70% ethanol and soap, and the blade/shaft cleaned with 70% ethanol. Disease was assessed on the flour via quantification of *F. graminearum* biomass and DON contamination.

DNA extractions were performed in triplicate, to account for sample variation across the large volume of collected grain, using the Qiagen DNeasy Plant Pro kit (∼20 mg each). *F. graminearum* biomass (hereafter *Fusarium* biomass) was determined by quantitative polymerase chain reaction (qPCR) on each replicate DNA extraction using previously reported protocols (Kemp et al., 2020). The qPCR was performed using the Juno and BiomarkHD instruments (Fluidigm, San Francisco, CA). The LinRegPCR program (v.1.0; Ruijter et al., 2009) was used to correct raw fluorescence values for variable amplification efficiencies by first determining a baseline, then estimating amplification efficiency for each sample individually, and computing a starting quantity. An arithmetic mean was calculated from four technical replicates per assay and then the geometric mean was computed from the three primer assays per organism (i.e., *F. graminearum* and wheat, primers and probes listed in Supplementary Table S2). To minimize error associated with variation in DNA extraction efficiencies, *Fusarium* biomass was expressed as the ratio of *Fusarium* DNA to wheat DNA. A final arithmetic mean was calculated on the three DNA extraction replicates for each field plot as an average. Lastly, the coefficient of variation was calculated and assessed as a measure of accuracy (Supplementary Figure S1).

Toxin analysis was performed in triplicate. Ground grain material (∼0.50 g) was extracted with 10 mL of acetonitrile-water (86:14 vol/vol) in a 50 mL Falcon tube with shaking for 15 min. Following centrifugation, a 5 mL portion of each extract was purified through a small column packed with 1.5 g of a mixture of silica gel C18, alumina, and activated charcoal (150:450:2.5). Then, a 2 mL aliquot of each purified extract was dried under a stream of air. Trimethylsilyl (TMS) derivatives were prepared as described previously (Whitaker et al., 2023). TMS derivatives of purified DON (0.3125–80 μg) were used to construct a standard curve for quantitation.

GC-MS analyses were performed on an Agilent 7890 gas chromatograph fitted with a HP-5MS column (30 m, 0.25 mm, 0.25 μm) and a 5977 mass detector as described previously (Whitaker et al., 2023). Similarly to the *Fusarium* biomass results, the coefficient of variation was assessed as a measure of accuracy (Supplementary Figure S1).

### 2.5 Bioinformatics

Sequence data were processed using *DADA2* (v.1.26.0) (Callahan et al., 2016) in R (v.4.2.2) (R Core Team, 2024) to determine amplicon sequence variants (ASVs). Primers were removed using *cutadapt* (v.1.18) (Martin, 2011) and default parameters were used for all other steps. After filtering, denoising, merging, and chimera check, the raw paired read count of experimental samples was reduced from 12,657,525 to 7,998,719. Non-fungal ASVs, including plant- and algal-origin and chimeras not screened by DADA2, represented < 0.5% of the remaining data and were removed in two steps. First, by using a BLAST search (-word_size 50, -perc_identity 60) to a manually curated database of full length ITS sequences for wheat, maize, and barley and the UNITE database (Kõljalg et al., 2013). Second, for sequences with neither high confidence matches to plants nor fungi, using a BLAST search to the NCBI nr database (Clark et al., 2016). Analysis of the negative controls revealed that two ASVs were present in two plates of the first round of PCR, one of which was unique across the dataset and the other which was not. No contaminants were present in the second round of PCR. Thus, we simply removed both ASVs from further analyses, resulting in 2580 ASVs. Putative taxonomy was assigned using the QIIME2 implementation (Bolyen et al., 2019) of a naïve Bayes classifier (classify-sklearn) (Bokulich et al., 2018) with the UNITE fungal ITS database (v10, Euk, RefS, dynamic) (Abarenkov et al., 2025). All taxonomic assignments were manually curated to replace instances of “*incertae sedis*” with “NA” that were otherwise unidentified at the genus level. Fungal richness (vegan v.2.6–4) (Oksanen et al., 2022) was calculated after quality filtering.

### 2.6 Statistical analyses

Statistical analyses were performed in R (v.4.4.1) and made substantial use of the *DADA2* (v.1.28.0), *phyloseq* (v.1.42.0) (McMurdie and Holmes, 2014), and *DESeq2* (v.1.38.3) (Love et al., 2014) packages. All data figures were created using *ggplot2* (v.3.4.1) (Wickham, 2016). The bioinformatics and statistical scripts, as well as experimental data, have been archived on Zenodo (Whitaker and Gdanetz, 2025).

#### 2.6.1 Community richness, structure, and variance partitioning analyses

To determine whether tissue type, site, host variety, sampling timepoint, and FHB disease status impacted fungal richness (number of unique taxa per sample) and community structure (dissimilarity in fungal communities between samples) in wheat, we constructed a series of models to test three sets of hypotheses (full ANOVA results in Supplementary Tables S3, S4). First, the impact of tissue type on fungal community richness and structure was tested as a stand-alone model predictor, due to the complexities inherent in sampling different plant tissue types over time. Second, models were constructed to test whether community structure was driven by site, variety, time, block, and their interactions as model predictors, for each tissue type separately. For the wheat heads, only the low disease heads at T5 were included in this model set—on the assertion that these were the “normal” head status in the field and to avoid partially nested variables during the analysis of timepoint. Lastly, a model was constructed for the wheat heads collected at the T5 timepoint to test whether community structure was driven by site, variety, disease status, block, and their interactions. In the second and third set of models, site, variety, and either time or disease status were tested as fixed effects, while block and associated interaction terms were tested as random effects. Fungal richness was analyzed as a univariate response, while community structure was analyzed as a multivariate response. To remove the dependence of variance on the mean in overdispersed sequencing count data (Warton et al., 2011), a variance stabilizing transformation (VST; in *DESeq2*) was applied to the ASV taxa matrix, with Euclidean distance then used to compute community dissimilarity between samples. In all cases, significant differences between treatments were tested using residual randomization in a permutation procedure (n = 999 permutations; RRPP package, v.1.3.1; Collyer and Adams, 2018) with Type 3 sum of squares. To visualize differences in communities, principal coordinates analyses (PCoA) were performed on the Euclidean distance of the VST-transformed ASV matrix.

To test whether the importance of host variety on mycobiome structure in wheat varied across tissue types, variance partitioning analyses using distance-based redundancy analyses (package *vegan*) were performed. Significance of treatment variables (site, timepoint, and either host variety or disease) was determined using conditional permutation tests (*n* = 999) for each tissue type separately.

#### 2.6.2 Source-sink analyses

To estimate the relative contribution of source communities to sink community composition, we used a microbial source tracking approach. Specifically, we employed the *FEAST* package which performs fast inference via expectation-maximization to estimate the contribution of different potential known and unknown sources to a target, or sink, community (Shenhav et al., 2019). Two types of source-sink analyses were constructed. First, we tested tissue-to-tissue transfer by assessing whether debris was a source to leaf sinks within the two relevant timepoints (T1 and T2) and whether leaves were a source to head sinks within the two relevant timepoints (T3 and T4). Second, we estimated how much the preceding timepoint was a source to subsequent timepoint sinks for all potential timepoint transfers (e.g., T1 source to T2 sink, T2 source to T3 sink, etc.) across all three tissue types independently. For the head tissues—transfer from the T4 source to the T5 sink was estimated for both low and high disease states. All source-sink proportion comparisons were made within a single field plot. Next, generalized linear models were constructed to test the importance of source type (known and unknown) across timepoints or disease states. Due to the bounded nature of the estimated source proportions (0,1), models were fit with a beta regression distribution (Cribari-Neto and Zeileis, 2010) in the *betareg* package (v.3.2.1; Zeileis et al., 2024). Significant differences among treatments were determined following the estimated marginal means method (package emmeans, v.1.10.5) (Lenth and Piaskowski, 2024), with *P*-values adjusted for Type 1 error using a Bonferroni correction.

#### 2.6.3 Differential abundance analysis

To identify individual taxa responsive to disease in T5 timepoint wheat heads, we performed a differential abundance analysis using *DESeq2*. Sequence abundances were modeled as the response using negative binomial generalized linear models. All relevant fixed main effects were included in the base model (site, variety, and disease status). Differential abundance across disease states was tested by comparing the base model to a model without disease status included (as in Wagner et al., 2016). Wald tests were used to estimate log2-fold changes in abundance for each ASV and significance determined at *P* < 0.05 after adjustment for Type 2 error using a Benjamini-Hochberg correction.

#### 2.6.4 Co-occurrence network analyses

Lastly, to identify individual taxa responsive to *Fusarium* ASV abundance and determine network stability overall, we performed co-occurrence network analyses using *SpiecEasi* package (v.1.1.3) (Kurtz et al., 2015). Networks were constructed for high and low disease heads at the T5 timepoint, as well as for maize debris (combined T1 and T2 timepoints)—which is a source of fusaria inoculum to winter wheat in the USA. ASVs unidentified at the rank of family or higher were not included in the analyses—except for a manual curation of unidentified taxa that might belong to the genus *Fusarium* (see Supplementary methods). Next, ASVs were filtered to a minimum abundance of 50 reads across subsamples. Network layouts were selected to balance readability while highlighting connections to *Fusarium* spp. nodes. Constructed networks were assessed for classic network statistics (degree, betweenness centrality, and closeness centrality, etc.) using functions from the *igraph* package (version 2.1.1) (Csardi and Nepusz, 2006). Then, hub taxa were identified as network nodes with high degree, betweenness-centrality, and closeness-centrality (as in Gdanetz and Trail, 2017).

## 3 Results

### 3.1 Agronomy and mycobiome overview

Yield was 17.0 kg ha^–1^ higher on average in Urbana compared to Ewing (*P* < 0.001), but there were no significant varietal differences (*P* = 0.29, Figure 1c; Supplementary Table S3). By contrast, DON and *Fusarium* biomass varied by variety (*P* = 0.0401, *P* = 0.0269, respectively) at the Ewing site, but not at the Urbana site (Figure 1c; Supplementary Table S3). The highest DON contamination ( > 1ppm) was detected at the Ewing site in the two varieties with resistance ratings of 5 and 8 (moderately susceptible and moderately resistant, respectively). Neither DON nor *Fusarium* biomass predicted yield (*P* = 0.40 and *P* = 0.33, respectively).

In the wheat phyllosphere and maize debris mycobiome, we identified 2,580 ASVs across the growing season. Most ASVs belonged to the Ascomycota and Basidiomycota phyla (66.8 and 29.9% respectively). The genera that accounted for the most sequencing reads included *Cladosporium* (18.5%), *Fusarium* (12.3%), *Vishniacozyma* (8.5%), *Alternaria* (8.1%), *Hannaella* (5.0%), and *Sporobolomyces* (4.9%). The top ten most abundant ASVs varied in their relative abundance across treatments (Supplementary Figure S2) and accounted for 53.0% of all sequencing reads. They included two *Cladosporium*, as well as a *Fusarium*, *Alternaria*, *Vishniacozyma*, *Colletotrichum*, *Sporobolomyces*, *Bullera*, *Papilotrema*, and one taxon unnamed at the genus level. The majority of ASVs were unique to a single tissue type (84.5%; 2,179 ASVs), though some were still shared across all three tissue types (3.8%; 99 ASVs; Supplementary Figure S3).

### 3.2 Community richness, structure, and variance partitioning

**FIGURE 2:**
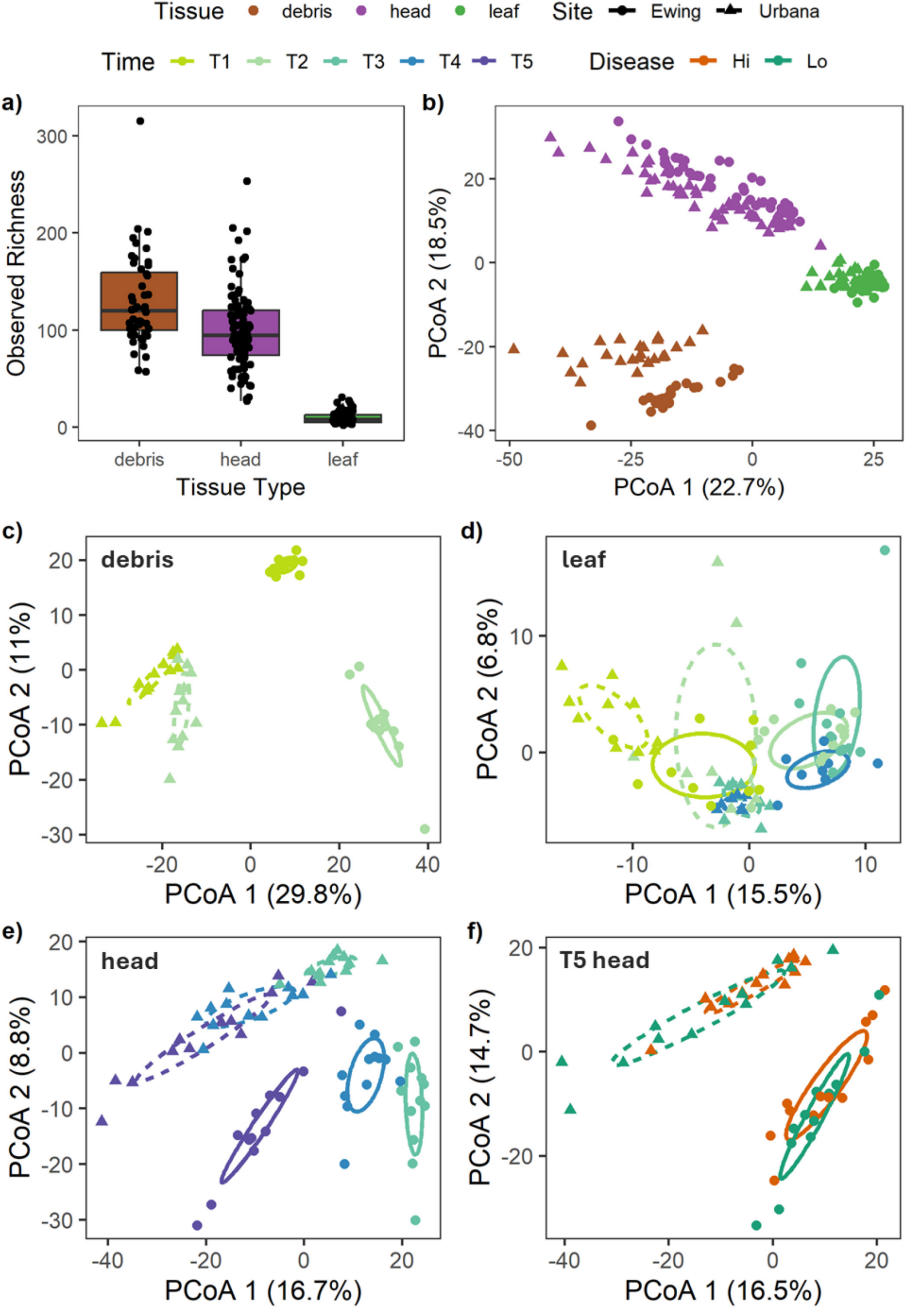
Community diversity varies as a function of tissue type, site, timepoint, and disease. Tissue type determines **(a)** observed fungal richness and **(b)** fungal community structure for all samples. Community structure also varies by site and timepoint of sampling for **(c)** debris, **(d)** leaf, and **(e)** head fungal communities, as well as by **(f)** site and disease status for the final collection (T5) of wheat heads. Each point represents an individual sample. For **(a)**, the boxplots show the median and interquartile range of the observed richness. For **(b–f)**, principal coordinates analyses (PCoA) are based on Euclidean distances of the variance stabilized taxa matrices and ellipses represent one standard error around the centroid for each treatment group. Colors denote tissue type **(a,b)**, timepoint (color shades): **(c–e)**, or disease status **(f)**.

Tissue type was a significant predictor of fungal richness (*P* = 0.001; Figure 2a), with leaf communities displaying the least richness relative to debris and head tissues. Similarly, mycobiomes were strongly structured by tissue type (*P* = 0.001; Figure 2b; Supplementary Table S3), with a clear visual separation in community structure along PCoA axes 1 and 2 by tissue type superseding differences between the two sites (Figure 2b).

Nevertheless, additional analyses by tissue type revealed that site, variety, and timepoint significantly affected community structure for debris and head tissues (significant 3-way interactions, both *P* = 0.001; Figures 2c,e; Supplementary Table S4). For leaf tissues, community structure was affected by significant two-way interactions between site and variety (*P* = 0.006), as well as between site and timepoint (*P* = 0.001), but there was no significant three-way interaction between site, variety, and timepoint (*P* = 0.274; Figure 2d; Supplementary Table S4). Visual assessment revealed differences in fungal community structure between the two sites in all tissue types (Figures 2c–e), as well as a clear temporal shift in the community structure results for debris and heads (Figures 2c,e), but a less clear temporal shift for leaf communities (Figure 2d). For the heads collected at the final timepoint, site, variety, and disease significantly impacted community structure (significant 3-way interaction *P* = 0.001; Figure 2f; Supplementary Table S4), with moderate shifts in the fungal communities visible in the PCoA (Figure 2f).

**FIGURE 3:**
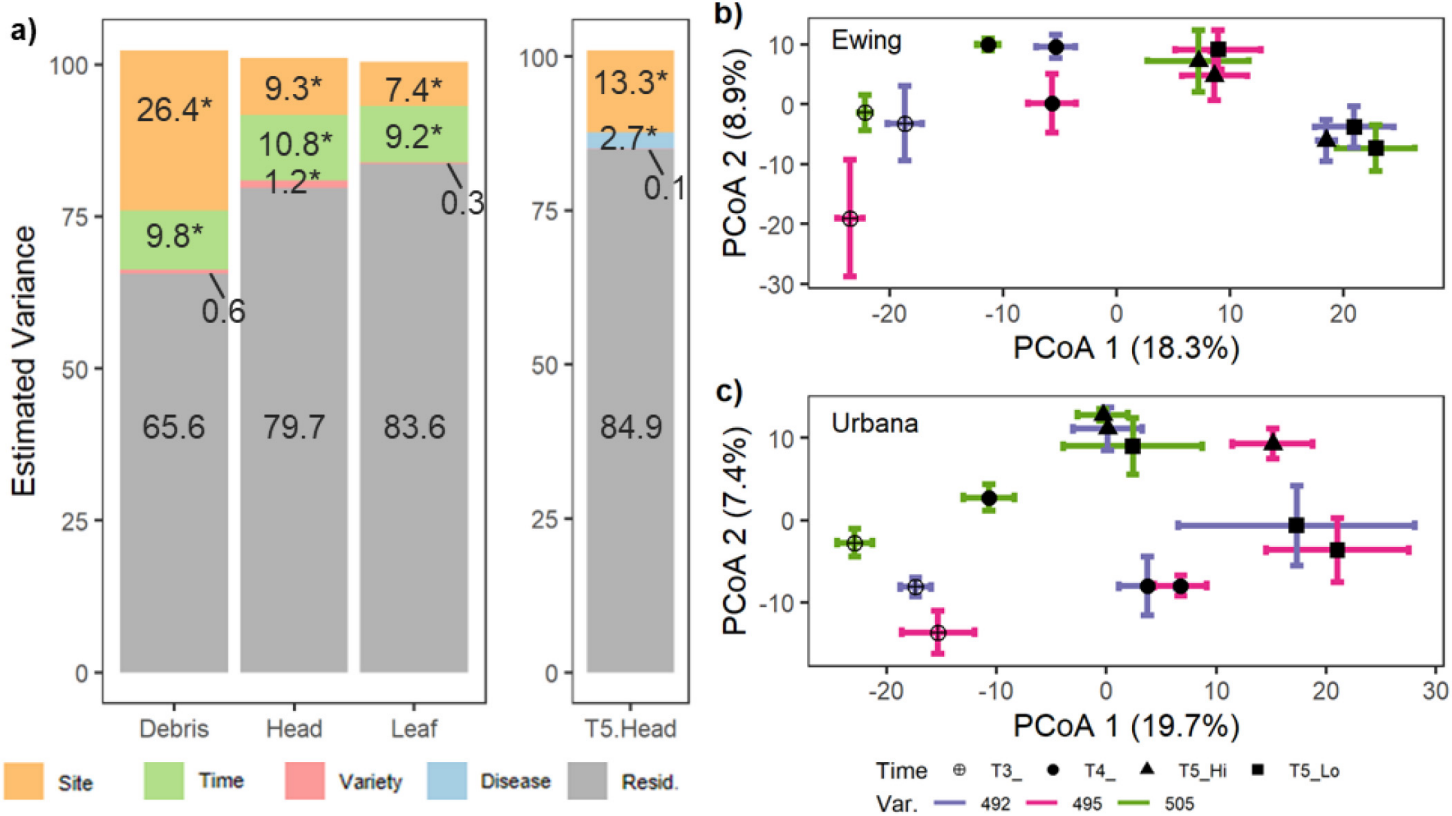
Host variety only explains variation in community structure in wheat heads. **(a)** Results from variance partitioning analysis describing the percentage of community structure explained by experimental treatments and unexplained residual variation for each tissue type and for the final collection of wheat heads (i.e., T5.Head). Asterisk (*) indicates a significant result by a conditional permutation test. For wheat heads in **(b)** Ewing and **(c)** Urbana, fungal community structure varies by AgriMAXX variety, timepoint, and disease. Each point represents the average location in ordination space for a treatment group, while error bars represent one standard error along the first and second axes. Principal coordinates ordinations are based on Euclidean distances of the variance stabilized taxa matrix.

Variance partitioning analysis revealed that the importance of treatments in explaining variation in the mycobiome differed across tissue types. Specifically, site and timepoint explained nearly equal conditional variation in fungal community structure in leaf and head tissues (7.4–10.8%; Figure 3a). However, site explained 2.5 times as much variation in community structure as timepoint for debris (26.4% vs. 9.8%, respectively; Figure 3a). Host variety only significantly explained variation in community structure in wheat heads (1.2%; Figures 3a–c), but not in debris or leaves (0.3–0.6%; Figure 3a). At both sites, varietal differences between wheat head communities tended to be stronger earlier in development (e.g., T3), than later (e.g., T5; Figure 3b,c). For the heads collected at the final timepoint (T5), disease and site significantly explained fungal community variation, with site explaining 5x as much (2.7% vs. 13.3%, respectively; Figure 3a). Across all tissue types, unexplained variation in fungal community structure predominated (66–85%; Figure 3a).

### 3.3 Source-sink analyses across tissues and timepoints

**FIGURE 4:**
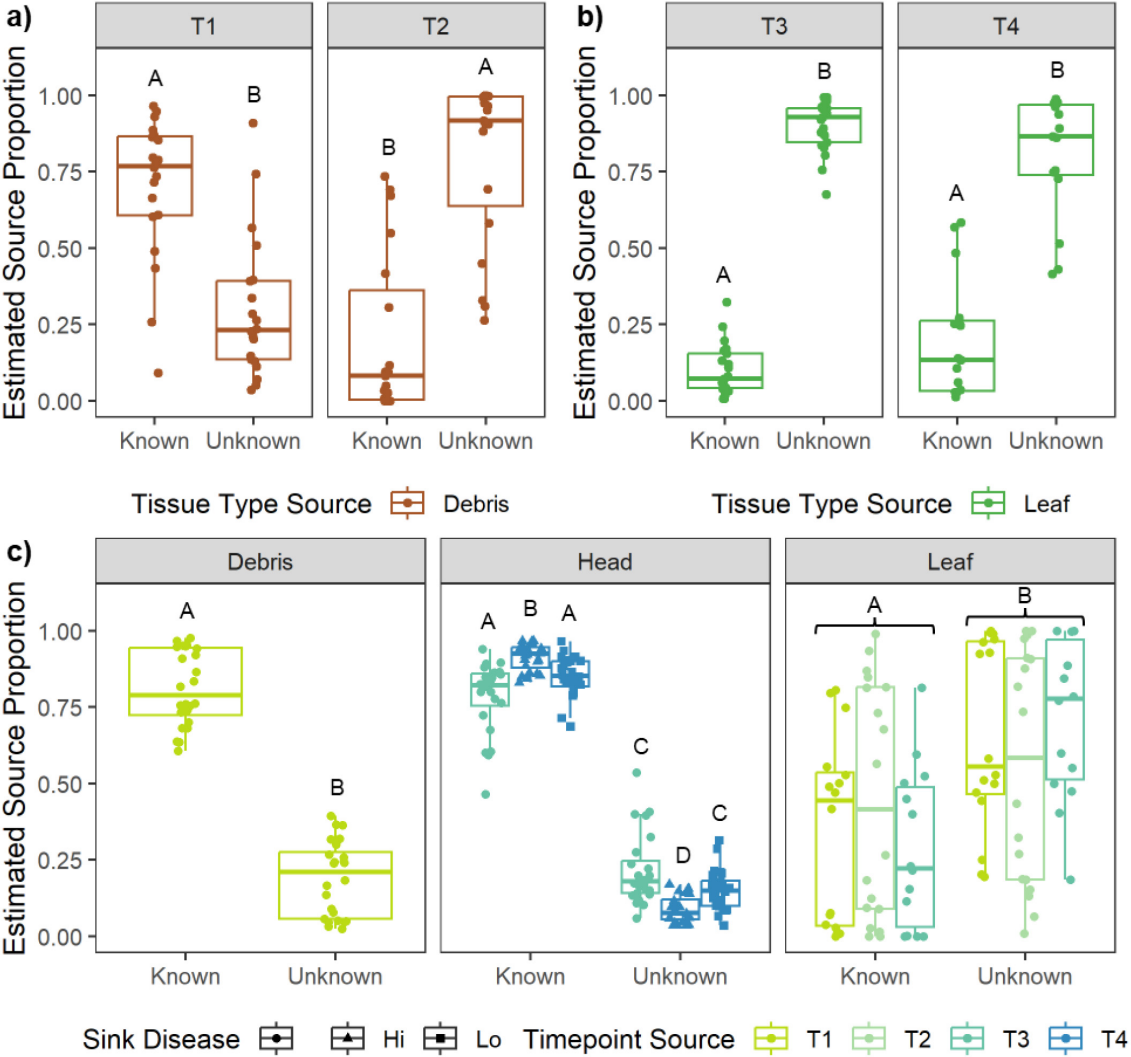
Estimated source proportions of fungal communities transferred **(a)** from debris sources to leaf sinks, **(b)** leaf sources to head sinks, and **(c)** from prior timepoints to subsequent timepoints. In all cases, the proportion of the tested sink community that can, or cannot be, explained by the tested source are referred to as “Known” or “Unknown,” respectively. For **(a,b)** different panels represent source-sink comparisons within the relevant developmental timepoints (**a** = T1 and T2; **b** = T3 and T4). For **c**) different panels represent source-sink comparisons within different tissue types (Debris, Heads, Leaves). For the Heads source-sink comparison, the proportion of the T5 sink explained by the T4 source is shown separately for each T5 disease status. Each point represents a single source-sink estimation within an experimental field plot. Boxplots depict the median, interquartile range, and 1.5x the interquartile range. Different letters indicate significant source differences based on *post-hoc* tests.

The source-sink analyses of tissue-to-tissue fungal transfer revealed that debris was a major source of fungi to wheat leaves during early development (i.e., T1), but not later (i.e., T2; Supplementary Tables S5, S6). Specifically, the estimated source proportion from debris to leaves was 0.70 on average before the over-wintering period compared to 0.20 after (Figure 4a). On the other hand, leaves were consistently a low source of fungi to wheat heads, both during heading and at flowering (T3 and T4; Supplementary Tables S5, S6), with average estimated source proportions of 0.10 and 0.20, respectively (Figure 4b).

Next, we used source-sink analyses to identify how much the preceding timepoint was a source to subsequent timepoints, for each tissue type. The preceding timepoint was a high source of fungi to the subsequent timepoint for both debris and head tissues, regardless of the preceding timepoint tested as a source or disease status of the heads tested as a sink (average source proportion = 0.78–0.91; Supplementary Tables S5, S6; Figure 4c). However, estimated marginal means analysis revealed that high disease heads during dough filling (T5) were a significantly greater recipient of fungi from flowering heads (T4), than were low disease heads (0.91 vs. 0.85 on average; Supplementary Table S6; Figure 4c). In other words, low-disease heads were colonized by a greater proportion of fungi from an unknown source and slightly lower proportions of fungi that had colonized during flowering. By contrast, early timepoint leaves were not a major source of fungi to later timepoint leaves (Supplementary Tables S5, S6), with average source proportion varying from 0.29 to 0.45 across the three timepoints tested (Figure 4c).

### 3.4 Differential abundance of fungi in response to FHB

**FIGURE 5:**
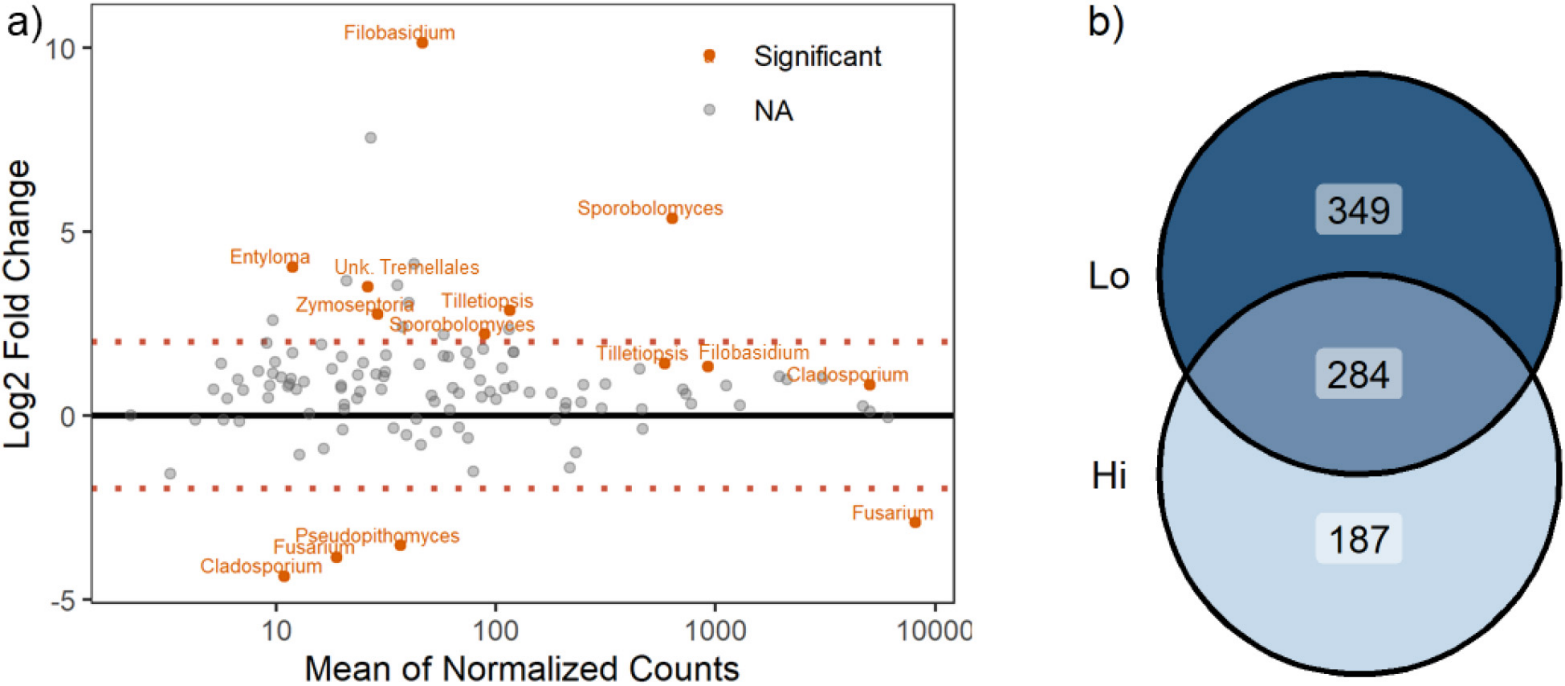
FHB disease alters fungal recruitment in T5 wheat heads. **(a)** The abundance of fungal taxa responsive to disease varies with their mean abundance. Positive values along the y-axis indicate enrichment under low disease, while negative values indicate enrichment under high disease. Taxa responding significantly are labeled (genus name) and denoted in orange. Dashed lines indicate a net log_2_ fold change of at least “2.” **(b)** Venn diagram of shared and unique fungi in T5 wheat heads. Lighter to darker shades indicate increasing ASV counts in the set.

Given the importance of the FHB-pathosystem in wheat, we used *DESeq2* to identify fungal taxa that were differentially enriched under high or low disease conditions (T5 timepoint). Only 14 ASVs were significantly differentially abundant according to disease state, after accounting for site and variety differences (Supplementary Tables S7; Figure 5a). Of these, most (10 ASVs) were enriched under low disease, with the remaining (4 ASVs) enriched under high disease. The taxa enriched in high disease heads all belonged to the Ascomycota, including two *Fusarium* (ASV2, ASV278), a *Pseudopithomyces* (ASV122), and a *Cladosporium* (ASV151). The identification of two enriched fusaria ASVs corroborated our visual selection for high disease symptoms in the field. However, some Fusarium infection was still identified in low disease heads (mean relative abundance of all Fusarium ASVs in low vs. high disease: 6.5% vs. 30.2%). Of the taxa enriched for low disease, most were basidiomycetous yeasts (8 ASVs) and included two *Filobasidium* (ASV11, ASV155), two *Sporobolomyces* (ASV27, ASV96), two *Tilletiopsis* (ASV31, ASV99), one *Entyloma* (ASV331), and one unknown Tremellales (ASV201). Two Ascomycota (ASV1:*Cladosporium* and ASV29:*Zymoseptoria*) were also enriched in low disease heads. Overall, more ASVs were unique to the low disease heads (42.6%) (Figure 5b) than were unique to the high disease heads (22.8%) (Figure 5b); though many ASVs were still shared between the two disease states (34.6%) (Figure 5b).

### 3.5 Co-occurrence networks

**FIGURE 6:**
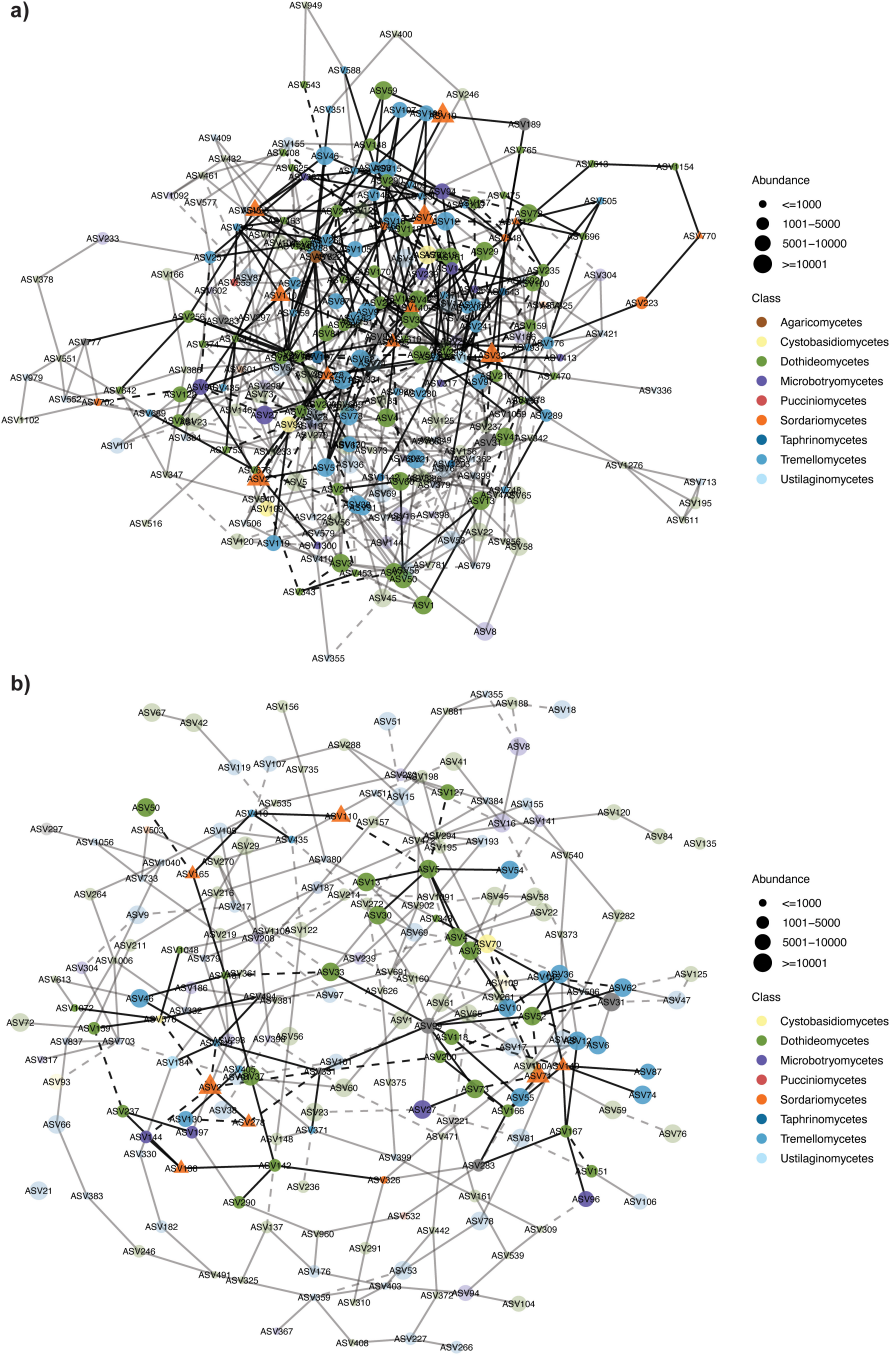
Microbial co-occurrence network of fungal taxa present in T5 wheat heads: **(a)** low disease or **(b)** high disease status. Nodes represent individual ASVs, colored by fungal Class, and size reflects approximate abundance. Edge line type reflects positive (solid) or negative (dashed) associations. Transparency of nodes and edges outside of 1st and 2nd degree connections to *Fusarium* spp. nodes was increased to 75%. Fusarium nodes are emphasized as triangles and are colored orange for being within the Class:Sordariomycetes.

Statistical comparison of co-occurrence networks indicated expected complexity based on sample type, with maize debris displaying highest complexity (Table 1, Supplementary Table S8 and Supplementary Figure S4), followed by the wheat head networks (Table 1, Supplementary Table S9, and Figure 6). As observed in other pathosystems, high-disease wheat head networks had reduced complexity relative to low disease head networks, as indicated by fewer nodes, edges, and hub taxa, as well as smaller average edge weight—suggesting reduced community stability (Figures 6a,b and Tables 1, 2).

**TABLE 1:** Network summary statistics.

Statistic	High disease	Low disease	Maize debris
# of nodes	198	255	354
# of edges	343	656	1445
Average edge weight	8.4	10.4	10.0
Min. node degree	1	1	1
Avg. node degree	3	5	8
Max. node degree	10	12	18
Hub Taxa	27	33	55
1st degree-*Fusarium* spp.	20	46	112
2nd degree-*Fusarium* spp.	34	97	184

**TABLE 2:** Hub taxa identified during network analysis.

Network	Node	Degree	Betweenness	Closeness
High disease	ASV2^+^: *Fusarium*	5	3774	2.56E-04
	ASV4: *Alternaria*	5	1496	2.52E-04
	ASV5: Unc. Didymellaceae	7	1224	2.29E-04
	ASV12: *Hannaella*	5	1948	2.15E-04
	ASV16: *Sporobolomyces*	4	2204	2.16E-04
	ASV27^+^: *Sporobolomyces*	4	2021	2.32E-04
	ASV33: *Alternaria*	4	1428	2.15E-04
	ASV69: *Bullera*	4	1565	2.32E-04
	ASV100: *Cercospora*	6	1998	2.11E-04
	ASV195: *Neosetophoma*	5	2139	2.43E-04
	ASV200: Unc. Phaeosphaeriaceae	5	1293	2.59E-04
	ASV239: *Leucosporidium*	5	4721	2.55E-04
	ASV272: *Alternaria*	4	1932	2.30E-04
	ASV349: *Dioszegia*	5	4362	2.59E-04
	ASV371: *Udeniomyces*	4	1070	2.13E-04
	ASV373: *Alternaria*	4	2031	2.16E-04
	ASV435: *Cryptococcus*	7	2023	2.24E-04
	ASV494: *Entyloma*	4	3954	2.55E-04
	ASV576: Symmetrospora	5	4405	2.55E-04
	ASV902: *Cladosporium*	4	1225	2.41E-04
	ASV1109: Unc. Mycosphaerellaceae	4	2947	2.46E-04
	ASV70*: *Symmetrospora*	7	4214	2.61E-04
	ASV99*^+^: *Tilletiopsis*	10	6149	2.62E-04
	ASV331*^+^: *Entyloma*	6	5592	2.62E-04
Low disease	ASV10: *Papiliotrema*	8	961	3.61E-04
	ASV21: *Hannaella*	7	2428	3.87E-04
	ASV28: *Papiliotrema*	7	3135	3.85E-04
	ASV37: Unc. Didymellaceae	9	1490	3.60E-04
	ASV69: *Bullera*	7	2028	3.83E-04
	ASV105: *Filobasidium*	7	1776	3.59E-04
	ASV130: *Papiliotrema*	8	1433	3.69E-04
	ASV135: *Neosetophoma*	8	4083	4.10E-04
	ASV141: *Rhodotorula*	7	3119	3.89E-04
	ASV160: *Ophiosphaerella*	10	2330	4.01E-04
	ASV197: *Sporobolomyces*	8	1992	3.79E-04
	ASV211: Unc. Phaeosphaeriaceae	8	2525	3.86E-04
	ASV221: *Pseudomicrostroma*	7	1766	3.82E-04
	ASV235: *Leptospora*	7	1203	3.68E-04
	ASV241: *Mrakia*	7	5343.5	4.12E-04
	ASV266: *Papiliotrema*	9	2148.5	3.66E-04
	ASV285: *Septoriella*	8	2478	3.69E-04
	ASV463: Unc. Phaeosphaeriaceae	8	1341	3.68E-04
	ASV466: *Microdochium*	10	1498	3.62E-04
	ASV643: Protomyces	9	1411	3.59E-04
	ASV963: Unc. Leucosporidiaceae	7	1377	3.76E-04
	ASV1142: *Taphrina*	7	1050	3.70E-04
	ASV1233: *Alternaria*	12	2913	3.82E-04
	ASV70*: *Symmetrospora*	9	2982	4.11E-04
	ASV167*: *Phaeosphaeriaceae*	11	4134	4.03E-04
	ASV193*: *Bullera*	10	5233.5	4.13E-04
	ASV371*: *Udeniomyces*	9	3013.5	4.00E-04
	ASV494*: *Entyloma*	11	5056.5	4.11E-04
Maize debris	ASV3: *Cladosporium*	14	4412	4.00E-04
	ASV4: *Alternaria*	14	1919	4.11E-04
	ASV14: *Fusarium*	11	4504	4.14E-04
	ASV17: *Vishniacozyma*	16	2212	4.09E-04
	ASV18: *Vishniacozyma*	14	2032	3.96E-04
	ASV19: *Fusarium*	10	1944	4.09E-04
	ASV28: *Papiliotrema*	14	3057	4.12E-04
	ASV31^+^: *Fusarium*	14	2978	4.02E-04
	ASV35: *Meyerozyma*	17	2334	3.94E-04
	ASV50: *Neosetophoma*	15	3233	3.97E-04
	ASV57: *Kurtzmaniella*	11	2511	4.12E-04
	ASV60: *Didymocyrtis*	16	2684	4.04E-04
	ASV78: *Vishniacozyma*	13	2352	3.95E-04
	ASV85: *Fusarium*	11	3270	4.12E-04
	ASV93: *Symmetrospora*	11	6545	4.30E-04
	ASV95: *Fusarium*	10	2883	4.05E-04
	ASV96^+^: *Sporobolomyces*	13	2311	4.07E-04
	ASV100: *Cercospora*	13	2564	3.95E-04
	ASV120: *Neosetophoma*	11	5057	4.30E-04
	ASV141: *Rhodotorula*	15	1613	4.11E-04
	ASV145: *Papiliotrema*	11	2644	4.09E-04
	ASV166: *Ophiosphaerella*	10	2988	4.07E-04
	ASV180: Unc. Dictyosporiaceae	11	2655	4.08E-04
	ASV190: *Phaeosphaeria*	12	1515	4.11E-04
	ASV191: *Hannaella*	14	2477	4.05E-04
	ASV194: Unc. Helotiales	11	4251	3.99E-04
	ASV204: *Clohesyomyces*	12	2798	4.08E-04
	ASV219: *Phaeosphaeria*	13	1902	3.93E-04
	ASV243: *Pseudocoleophoma*	15	3665	4.01E-04
	ASV246: *Aureobasidium*	11	1448	4.27E-04
	ASV254: *Hannaella*	10	2908	4.05E-04
	ASV260: *Schizothecium*	14	2486	4.07E-04
	ASV269: *Edenia*	10	1451	4.03E-04
	ASV274: *Vishniacozyma*	17	2787	4.08E-04
	ASV308: *Pleosporaceae*	12	2340	4.07E-04
	ASV333: *Filobasidium*	11	2220	4.01E-04
	ASV393: *Hannaella*	12	2141	3.93E-04
	ASV421: *Vishniacozyma*	12	2620	3.97E-04
	ASV498: *Keissleriella*	10	1990	3.99E-04
	ASV580: *Vishniacozyma*	14	2416	4.19E-04
	ASV637: *Zymoseptoria*	13	2410	4.11E-04
	ASV677: *Saitozyma*	11	1428.5	3.98E-04
	ASV697: *Populomyces*	10	3319	4.03E-04
	ASV798: *Papiliotrema*	11	4330	4.13E-04
	ASV1227: *Fusarium*	11	2916	3.93E-04
	ASV12*: *Hannaella*	14	5672	4.30E-04
	ASV37*: Unc. Didymellaceae	18	6644	4.15E-04
	ASV59*: *Neoascochyta*	16	6757	4.28E-04
	ASV66*: *Hannaella*	18	5955	4.13E-04
	ASV209*: *Colacogloea*	15	8687	4.27E-04

ASVs listed are in 75th percentile for all network statistics, unless denoted by asterisk (*) which indicates 95th percentile. ASVs denoted by (+) were identified as significantly associated with one of the disease states in the DESeq2 analysis. “Unc.” indicates unclassified at the family level.

Four hub taxa (ASV69:*Bullera*, ASV70:*Symmetrospora*, ASV371:*Udeniomyces*, ASV494:*Entyloma*) were shared between networks of the two disease states (Table 2). Three hub taxa each were shared between the maize debris and high-disease (ASV4:*Alternaria*, ASV12:*Hannaella*, ASV100:*Cercospora*) or between the maize debris and low-disease (ASV28:*Papiliotrema*, ASV37:Didymellaceae, ASV141:*Rhodotorula*) networks, respectively. No hub taxa were shared across all three networks (Table 2). The *DESeq2* analysis identified 14 ASVs as significantly different between disease status of heads; four of these were also identified as hub taxa in the high disease network (including ASV2:*Fusarium*) and two in the debris network (Table 2).

The greatest number of *Fusarium* spp. nodes (26 ASVs) were present in the maize debris network (Supplementary Table S8), with six *Fusarium* nodes shared with at least one of the head networks (Supplementary Table S10). Four *Fusarium* spp. nodes were present in both wheat head networks and two *Fusarium* sp. nodes (ASV2, ASV278) were shared across all three networks (Supplementary Tables S9, S10). There were similar numbers of unique ASVs negatively associated with *Fusarium* spp. nodes in both head networks, with taxonomic assignments to Basidiomycota (ASV331:*Entyloma*, ASV94*Sporobolomyces*, ASV144: *Sporobolomyces*, ASV93:*Symmetrospora*, ASV576:*Symmetrospora*) and Ascomycota (ASV118:*Parastagonospora*). Taxa that positively co-occurred with *Fusarium* nodes in both disease states included: *Alternaria* (ASV431, ASV1154), *Ophiosphaerella* (ASV166, ASV294), *Papiliotrema* (ASV88, ASV106, ASV145, ASV266, ASV362), *Phaeosphaeriaceae* (ASV167), *Sporobolomyces* (ASV96, ASV144), and *Vishniacozyma* (ASV74, ASV87, ASV289, ASV410). Phylloplane yeasts; *Vishniacozyma*, *Aureobasidium*, *Filobasidium*, *Hannaella*, and *Occultifur* were frequently associated with *Fusarium* spp. in maize debris networks and rarely found in head networks (Supplementary Table S11). When these genera were detected in heads, it was in low-disease status samples, mirroring the *DESeq2* analysis.

## 4 Discussion

Inter- and intraseasonal transfer of microbiota between tissues was a critical driver of mycobiome structure in the wheat phyllosphere over the course of development. Specifically, we found that maize debris from the previous growing season was a major source to wheat leaf mycobiome composition during early development (T1:tillering), but that this crop-to-crop microbial transfer rapidly diminished after the overwintering period. In addition, despite the shared commonalities between wheat leaves and heads (i.e., both are aboveground, photosynthetic tissues emerging from stems), the average source proportion from leaves to heads was less than 20% with the unknown source proportion making up the remainder. Lastly, pathogenic *Fusarium* ASVs were identified as important hubs in the microbial networks of both wheat heads and maize debris, as well as an enriched component of high disease head mycobiomes—with concomitant restructuring of other presumptive beneficial, commensal, and pathogenic ASVs in the community networks. Ultimately, our results may be used to develop microbial-based disease prevention or plant growth promoting strategies in the wheat phyllosphere over the course of development.

Plant pathologists have known for decades that debris left behind in the field from previous crops can be a source of pathogen inoculum in the succeeding growth season (Smith and White, 1988; Cotten and Munkvold, 1998; Stack, 2003). However, contribution of debris microbiota to non-pathogenic members in the phyllosphere microbiome has remained unresolved. Here, we showed that the proportion of the winter wheat leaf mycobiome sourced from maize debris was about 70% on average when the plants were young and the maize debris only recently senesced. Over the winter, the debris mycobiome composition itself changed substantially (Figure 2), likely due to physical and nutritional changes associated with the decomposing tissue (Gregorich et al., 2017; Wijas et al., 2025). The ultimate result was decreased cross-colonization in maturing wheat plant leaves. The role of maize-derived microbiota in colonizing early-development wheat raises interesting questions about interseasonal legacy impacts and the ability of crops to resist diseases later in life. By contrast, the unknown source of fungi on early wheat leaves (Figure 4a) could be due to a number of factors, including sourcing from the seed microbiome or the impact of conventional seed treatments used in this region (Arnault et al., 2024). Recent research has highlighted the importance of microbiome composition on young plants for developing adequate immunocompetence and gatekeeping harmful, opportunistic members of the microbiome (Chen et al., 2020; Pfeilmeier et al., 2021; Paasch et al., 2023). In fact, carefully controlled priority effect (i.e., “order of arrival”) experiments have indicated that which microbes colonize plant tissues first can dictate disease resistance outcomes, irrespective of microbiome composition at the actual timing of pathogen infection (Leopold and Busby, 2020). Therefore, future research should consider the impact of management practices, such as crop rotation series and conventional farming strategies, on the microbiome and its relation to host immune development and disease resistance.

By contrast, the leaf mycobiome never served as a major source of fungi to the wheat head mycobiome. This result has important implications for determining the optimal developmental window for novel biocontrol applications—biocontrol strains or consortia applied to leaves prior heading may not effectively suppress grain diseases if the mode of action requires physical contact or ephemeral secreted molecules. The study design tested here does not allow direct quantification of the primary source of colonizers to wheat heads. However, there is qualitative evidence of colonizer origin from examination of the core mycobiome. Fifty-six fungi were identified as part of the core mycobiome using the definition from Shade and Stopnisek (2019) which relies on abundance and occupancy criteria. Of these core taxa, many were highly abundant across both maize debris and wheat head sample types (Supplementary Figure S5), despite the different timepoints sampled. It is possible that some fungi colonizing heads represented transient environmental fungi, or were sourced from debris, as is known for *Fusarium* (Miller et al., 1998), or that they came from another unmeasured source, such as soil (Grady et al., 2019), neighboring vegetation or fields (Whitaker et al., 2022), or another untested wheat tissue such as stems or roots (Gdanetz and Trail, 2017). Wheat heads are the physical habitat where direct competition between microbiota and *Fusarium* spp. can occur. Thus, a greater understanding of where wheat head-colonizing microbiota originate in the environment will be crucial to develop novel microbe-based intervention strategies for disease management.

Interestingly, the variation partitioning analysis revealed that the conditional impact of host variety on the mycobiome was only significant in wheat heads, but not in wheat leaves or maize debris. We selected winter wheat varieties which varied in their resistance to FHB, with ratings ranging from moderately susceptible to moderately resistant. Complete genetic resistance is not currently available for FHB, which is a disease governed by multiple host resistance loci of small effect (Miedaner et al., 2023). Cumulative efforts over the past 30 years to breed for FHB resistance may have indirectly altered the host’s interaction with its native microbiome in a developmental stage- (VanWallendael et al., 2022) or tissue-dependent manner (Wipf and Coleman-Derr, 2021). One recent study in maize showed that quantitative trait loci for disease resistance introgressed into a disease susceptible background altered both the bacterial and fungal leaf communities, but the genetic traits were ultimately not found to be a reliable predictor of community structure across fields due to gene-by-environment interactions (Wagner et al., 2020). Here, the selected varieties varied in many traits and were not introgressed for FHB resistance; however, we also detected evidence for a genetics-by-environment effect on disease and mycobiome structure. Overall, the Ewing site experienced much higher disease intensity, with only the AgriMAXX 505 variety (the most resistant variety) achieving DON levels below the US food safety advisory limit of 1 ppm (Figure 1c). The community structure for AgriMAXX 505 also showed the greatest community dissimilarity between the low and high disease states in Ewing (Figure 3b). Consequently, while variety likely influences the pathogen-microbiome interaction and disease, the specific outcomes may be dictated by environment and other aspects of host biology, such as the timing and tissue of pathogen infection.

The impact of disease on wheat head mycobiomes was apparent across multiple metrics of community diversity. For example, the number of unique fungal taxa was greatly reduced in high disease heads. A continental screen of wild *Arabidopsis thaliana* leaves showed similarly reduced microbial diversity in conjunction with foliar disease loads (Karasov et al., 2024). It is possible that *Fusarium* infection reduced resource and habitat quality in the phyllosphere due to host tissue compositional changes (e.g., necrotrophy; Bushnell et al., 2003). Alternatively, low disease heads may have been better able to resist *Fusarium* infection due to microbial recruitment in the face of disease pressure (i.e., “cry-for-help” phenomenon; Rolfe et al., 2019), as has been demonstrated in the rhizosphere with plant root exudates (Liu et al., 2021; Liu S. et al., 2023). However, mechanisms for microbial recruitment in the phyllosphere remain less clear, due to the different media (air vs. soil), microbial source origins, and colonization mode. In one recent study, phyllosphere recruitment of beneficial *Lactobacillus* and *Aspergillus* was linked to a metabolic host defense response in the face of false smut disease on rice panicles (Liu X. et al., 2023). Here, low disease T5 heads were colonized by larger proportions of fungal taxa from an external, unknown source than were high disease T5 heads (Figure 4c). This increased source proportion likely represents both increased numbers of unique taxa colonizing the heads (Figure 5b), as well as potential abundance shifts (Figures 5a, 6a). Determining the molecular mechanisms by which plants recruit beneficial phyllosphere taxa could represent new breeding targets for FHB that have yet to be fully explored.

Co-occurrence network analysis of microbial communities can provide information on community stability overall, as well as on candidate taxa that respond to experimental conditions. Here, the network analysis showed reduced connectivity and complexity in the high relative to low disease networks, which has been observed in other pathosystems (Agler et al., 2016; Runge et al., 2023), and is often an indicator of reduced community stability (reviewed in Kajihara and Hynson, 2024). Additionally, the networks identified taxa that positively (e.g., *Vishniacozyma*, *Cercospora* spp.) or negatively (e.g., *Parastagonospora*, *Symmetrospora*, *Entyloma* spp.) co-occurred with *Fusarium* spp. in debris and wheat heads—which could be useful targets for biocontrol or whole microbial community management. For example, *Parastagonospora nodorum* is the causal agent of Septoria nodorum blotch and negatively co-occurred with *Fusarium* in head networks, indicating possible competitive exclusion. *P. nodorum* infects wheat heads by manipulating plant defense mechanisms (Kariyawasam et al., 2023). Thus, artificial manipulation of host defense mechanisms to mimic *P. nodorum* infection could lead to novel disease intervention strategies for FHB. Alternatively, there are many other modes of action for biocontrols (Schöneberg et al., 2015; Saravanakumar et al., 2017) which could be tested using *in vitro* or *in planta* assays for the network-identified taxa, including: competition for niche space, inhibition of growth due to secreted and/or volatile secondary metabolites, parasitism of the pathogen, and biofilm formation (Agirman et al., 2023).

Furthermore, the differential abundance analysis highlighted the potential role of yeasts in association with low FHB states. Over the last decade, phyllosphere yeasts have increasingly emerged as candidate biocontrols due to mounting evidence that they are both taxonomically and biochemically hyper-diverse (Gouka et al., 2022a). Examples of commercial products exist for specialty crops (e.g., BlossomProtect, YSY Agro) with stimulation of systemic acquired resistance pathways as the key mode of action (Zeng et al., 2023). At non-commercial scales, *Sporobolomyces* and *Aureobasidium* spp. isolated from wheat demonstrated inhibitory activity toward several wheat stem pathogens (Wachowska and Borowska, 2014). Members of these genera, as well as *Metschnikowia*, *Rhodotorula*, and *Vishniacozyma* yeasts, also inhibited *F. graminearum* growth *in vitro* (Gouka et al., 2022b). Similarly, a *Papilotrema flavescens* strain (formerly *Cryptococcus*) has shown efficacy against FHB in greenhouse trials (Schisler et al., 2015). Yeasts may also play a role in environmental management of *Fusarium* inoculum via competition on crop debris. For example, *Vishniacozyma* yeasts were shown to be negatively correlated with *Fusarium* spp. in maize debris (Cobo-Diaz et al., 2022). Some yeasts also have saprophytic lifestyles in soil and debris (Mašínová et al., 2018) and could thus speed decomposition and reduce over-winter inoculum potential. Further research is needed to understand the role of yeasts as potential biocontrols of FHB *in planta*, or as applications to debris in the field.

In conclusion, our results demonstrate that crop-crop transfer of fungi between seasons, as well as tissue-tissue transfer within seasons, are major drivers of community dynamics in the phyllosphere microbiome and potential indicators of disease outcomes in an FHB pathosystem. Microbiome-based disease intervention strategies are still in their infancy, but investment in the biologicals industry is rapidly advancing (Lau et al., 2022), as is enhanced awareness of microbial roles in farm management strategies and in modern breeding approaches (Compant et al., 2024). Here, our work showcases multiple potential research and development pathways for harnessing the microbiome to either improve plant performance or manage *Fusarium* spp. and their associated mycotoxins in wheat, including identifying: basidiomycetous yeasts as enriched under low disease conditions, specific fungal taxa that are correlated with *Fusarium* spp. in wheat heads and maize debris, and that biocontrol applications to wheat leaves are unlikely to manage pathogen spread in wheat heads. The next frontier in this research field will be tests of microbe-microbe interactions under controlled conditions, as well as assessments of impact on key crop performance criteria such as immune response, disease reduction, and yield.

## Data Availability

The datasets presented in this study can be found in online repositories. The names of the repository/repositories and accession number(s) can be found at: https://www.ncbi.nlm.nih.gov/, PRJNA1297200; https://doi.org/10.5281/zenodo.17122469, Zenodo 17122469.
