# Supplementary material for: Wheat mycobiome dynamics driven by interseasonal crop-crop transfer and Fusarium head blight
Source: Front Microbiol. 2026 Mar 4;17:1778987. doi: 10.3389/fmicb.2026.1778987 (PMC12996221; doi:10.3389/fmicb.2026.1778987)

Supplementary Material

**Supplementary Methods**

***Plot sampling strategy***

We sampled from eight points total per plot at each field site, to cover the entire length of the plot. However, the width of the plots varied by site, with two- 10 ft planter passes in Ewing and three- 10 ft planter passes in Urbana. To adequately sample the width of the plots, four points were sampled from each half of the plot, according to the design of the field plots by site. All samples were combined into one sterile plastic bag according to sample type and time point of the collection.

***Taxonomic curation for network analysis***

For the network analysis, an additional taxonomic curation step was taken to identify all possible *Fusarium* spp. Specifically, for the ASVs that were classified either as unknown Fungi, unknown Ascomycota, unknown Sordariomycetes, or unknown Hypocreales (160 ASVs total), their sequences were BLASTed against a restricted search of the 'nr' nucleotide repository for taxid match ‘Fusarium’. There were eight ASVs with either a high or moderate match to *Fusarium* (high query coverage and high percent identity [>=95%]). These eight ASVs were then BLASTed a second time using an unrestricted query. Only three were matches to *Fusarium* spp. in the unrestricted search (ASV39, ASV83, ASV1175) and were thus reassigned as *Fusarium* at the genus level in the network analysis.

**Supplementary Figure 1 – Deoxynivalenol (DON) mycotoxin correlates with *Fusarium graminearum* (Fg) biomass.** Each point represents a single sampled field plot, while point size displays the coefficient of variation (CV) for technical replicates of a) DON and b) Fg. Black line shows correlation with gray bar indicating the standard error. Three plots had one replicate DNA extraction fail to amplify across all three *F. graminearum* assays, so average Fg biomass was calculated from the remaining two DNA extraction replicates. One plot had two replicate DNA extractions fail, so Fg biomass was assigned at the level of the only successfully amplified replicate (which had a low *Fusarium* load and was probably near the limit of detection).


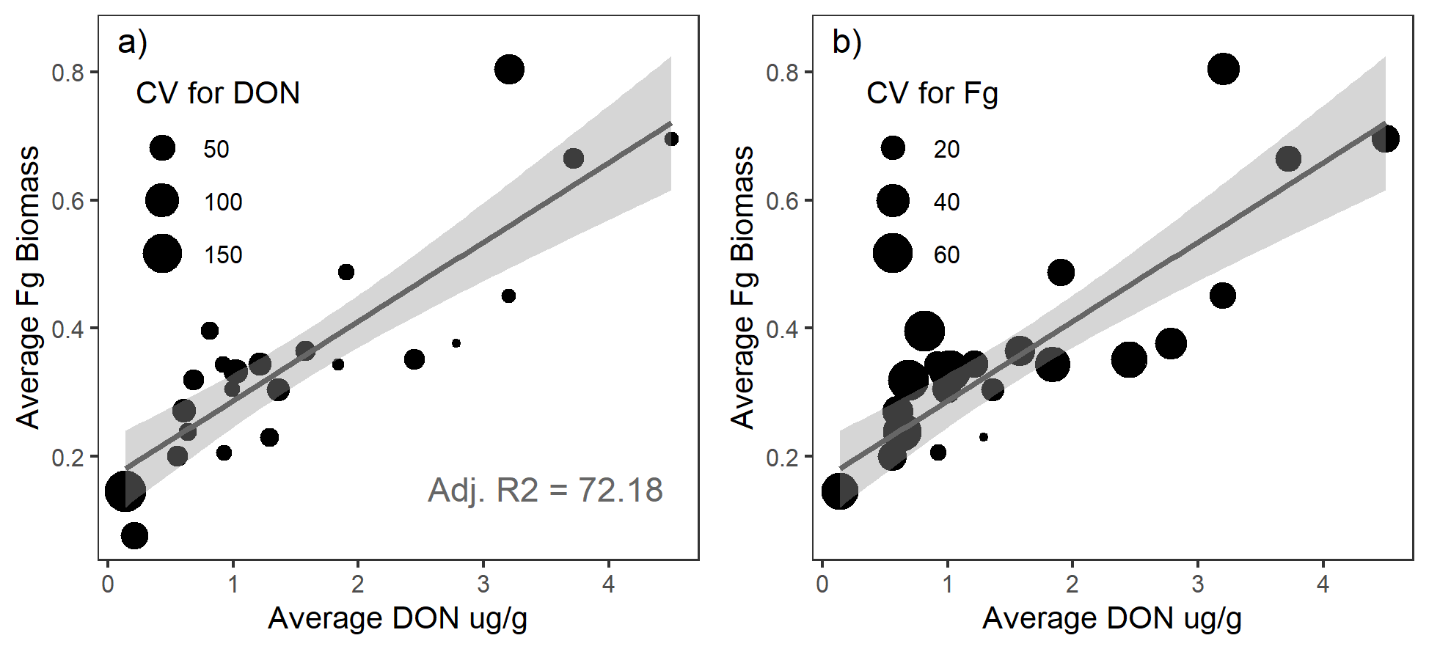


**Supplementary Figure 2 – Relative abundance of top ten most abundant ASVs** by read count, depicted across a) tissue types, b) sites, c) AgriMAXX varieties, and d) timepoints, with all other taxa grouped into “Other”. ASV1:*Cladosporium*; ASV2:*Fusarium*; ASV3:*Cladosporium*; ASV4:*Alternaria*; ASV5:Didymellaceae; ASV6:*Vishniacozyma*; ASV7:*Colletotrichum*; ASV8:*Sporobolomyces*; ASV9:*Bullera*; ASV10:Papilotrema.


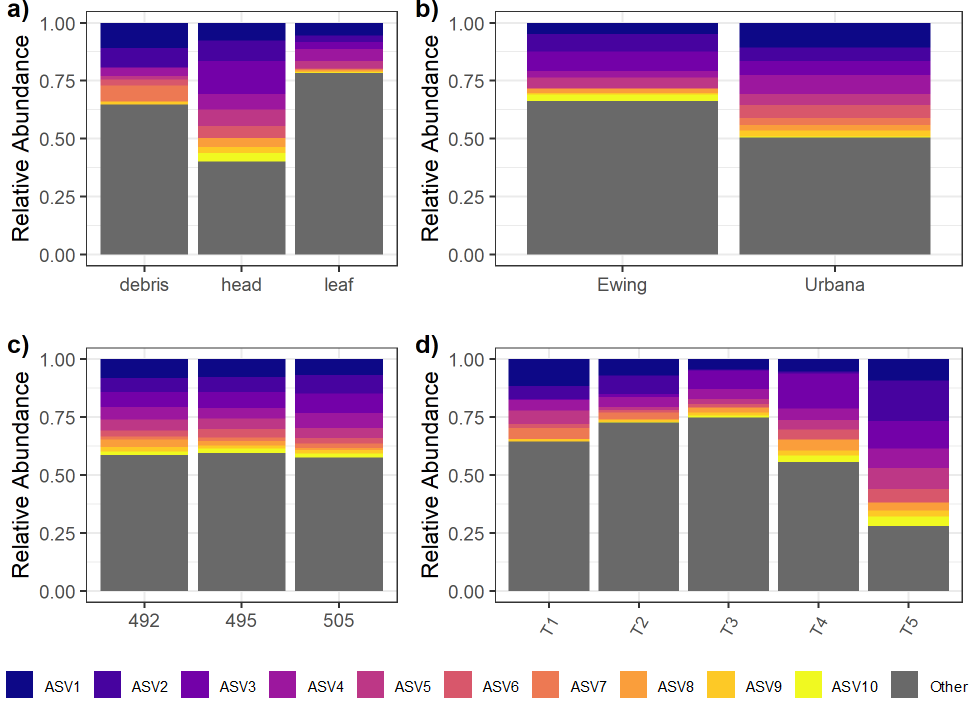


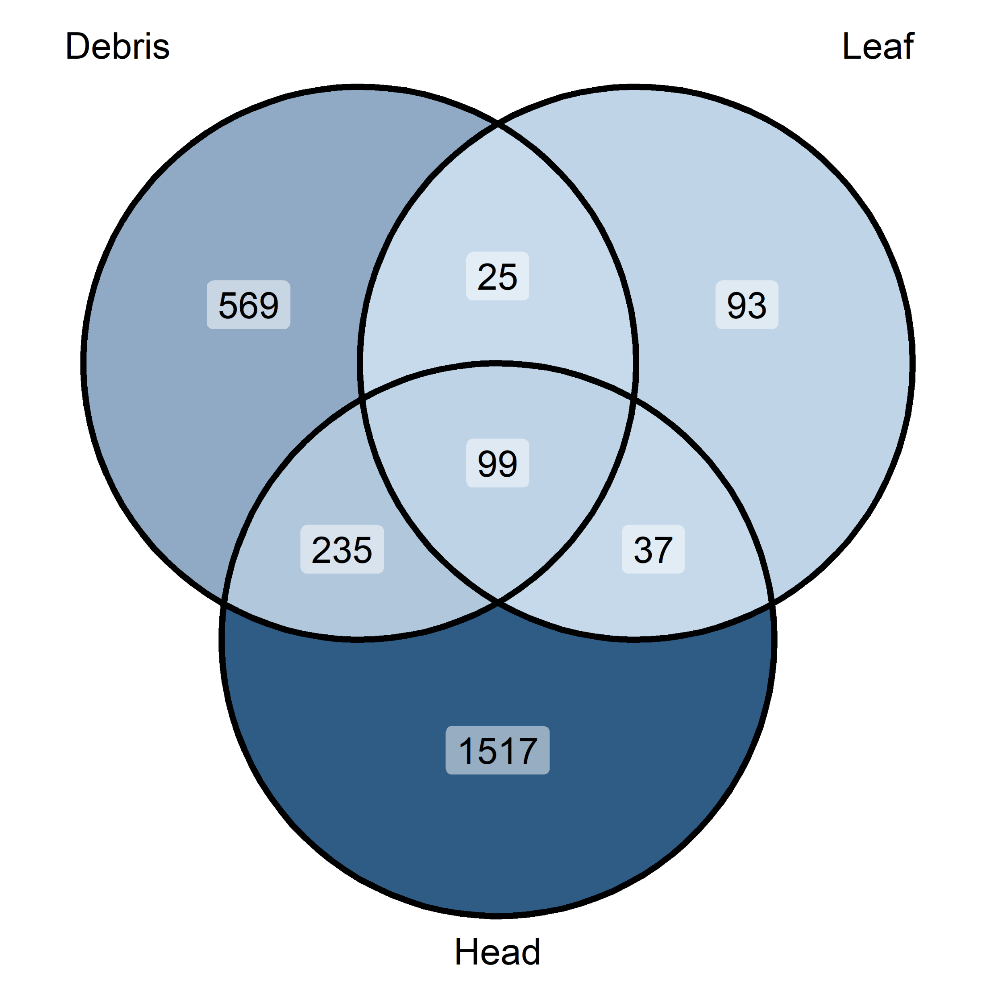
**Supplementary Figure 3 – Venn diagram of shared and unique ASVs across tissue types.** Lighter to darker blue shades indicate increasing ASV counts in the set. Data represents all samples grouped together.


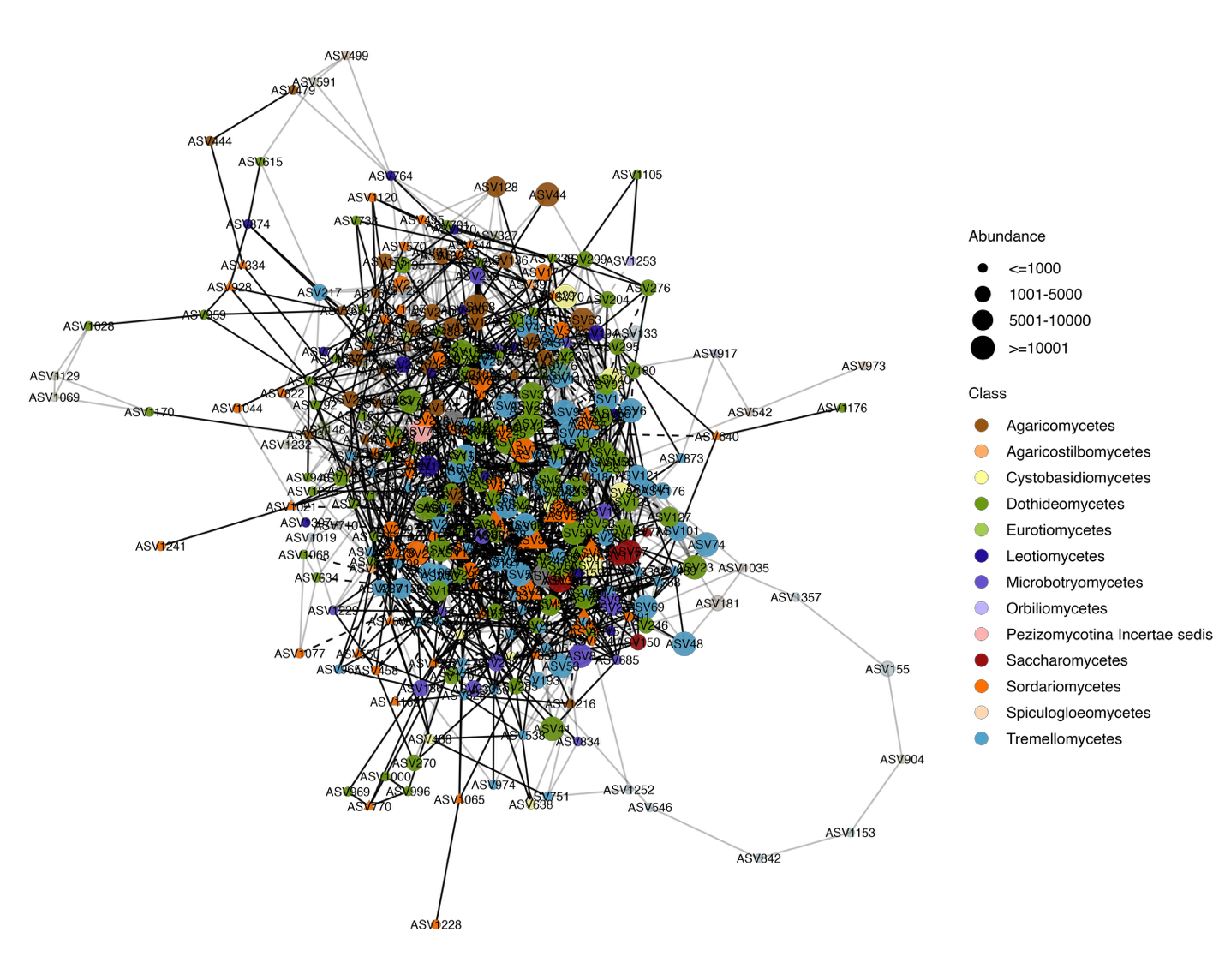
**Supplementary Figure 4 – Microbial cooccurrence network of fungal taxa present in corn debris collected from Illinois.** Nodes represent individual ASVs, colored by Class, and size reflects approximate abundance. Edge colors reflect positive (solid) or negative (gray) associations.

**Supplementary Figure 5 – Core mycobiome.** A) Fifty-six core fungi were identified using the Shade & Stopnisek (2019) method for a 3% increase in Bracy-Curtis similarity across sample types. b) Stacked bar plot showing the total read count of each core fungi colored by sample type.


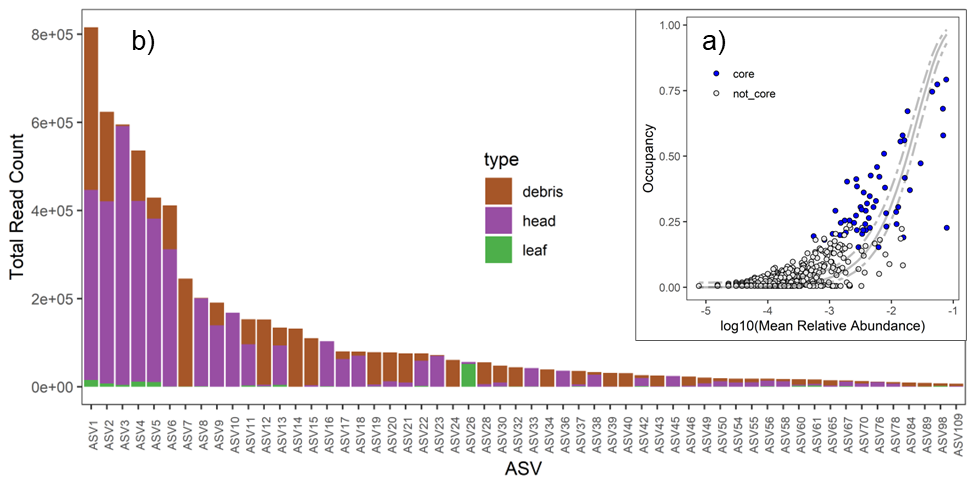

Supplement: Supplementary file 1 [file Data_Sheet_1.docx]
